# *Notes from the Field:* Exported Case of Sin Nombre Hantavirus Pulmonary Syndrome — Israel, 2017

**DOI:** 10.15585/mmwr.mm6740a5

**Published:** 2018-10-12

**Authors:** Aaron Kofman, Galia Rahav, Del Yazzie, Herman Shorty, Hayley D. Yaglom, Dallin Peterson, Melissa Peek-Bullock, Mary J. Choi, Anat Wieder-Finesod, John D. Klena, Heather Venkat, Cheng-Feng Chiang, Barbara Knust, Marlene Gaither, Matthew Maurer, Donald R. Hoeschele, Stuart T. Nichol

**Affiliations:** ^1^Epidemic Intelligence Service, CDC; ^2^Division of High-Consequence Pathogens and Pathology, National Center for Emerging and Zoonotic Infectious Diseases, CDC; ^3^Sheba Medical Center, Tel Hashomer, Israel; ^4^Sackler Medical School, Tel Aviv University, Israel; ^5^Navajo Department of Health, Window Rock, Arizona; ^6^Arizona Department of Health Services; ^7^Utah Department of Health; ^8^Nevada Department of Health and Human Services; ^9^Northern Arizona Healthcare, Flagstaff, Arizona; ^10^Coconino County Public Health Services District, Flagstaff, Arizona; ^11^National Park Service, Washington, DC.

In November 2017, CDC confirmed Sin Nombre virus (SNV) infection in a previously healthy man aged 47 years who was admitted to a hospital in Israel. The patient had traveled with his family on vacation to the southwestern United States (Arizona, Nevada, and Utah) during October 3–9, 2017. During this time, he and his family hiked and biked the southern rim of the Grand Canyon and Zion National Park and took a guided tour through Antelope Cave. On November 7, approximately 3 weeks after his return to Israel, he was hospitalized with fever, cough, and shortness of breath requiring bilevel positive airway pressure. A chest radiograph indicated diffuse reticulonodular infiltrates with consolidations at the right costophrenic angle and in the retrocardiac space. Based upon the patient’s travel history and clinical findings, hantavirus pulmonary syndrome was suspected. A blood specimen collected on November 9 tested positive for SNV using nested reverse transcription–polymerase chain reaction; he had an immunoglobulin M titer of ≥1:6,400 and an immunoglobulin G titer of ≥1:6,400. Hantavirus pulmonary syndrome has a mortality rate of approximately 36%.* The patient was treated with supportive care and discharged from the hospital on November 19. No illness was reported in any family member who traveled with him.

SNV is a species of hantavirus that is transmitted to humans primarily through contact with the infected urine or droppings of a deer mouse (*Peromyscus maniculatus*). Deer mice are present throughout most of the continental United States, but transmission is most common in the “Four Corners” region (Arizona, Colorado, New Mexico, and Utah) (*1*–*3*). The average incubation period is 1–5 weeks after exposure. There is no human-to-human transmission of SNV, and clustering of cases is uncommon. The patient did not report any known contact with rodents, rodent nests, or rodent droppings either in his places of lodging or during the course of his recreational activities.

An environmental investigation conducted by the Arizona Department of Health Services and the Utah Department of Health did not find any evidence of rodent infestation at the inns and hotels where the patient and his family stayed during their travels. In Antelope Cave, evidence of rodent burrowing was identified in areas around the canyon entrances as well as at the juncture of the canyon floor and walls. In addition, tour guides are known to throw sand from the canyon floor into the air to better illuminate the sunlight beams entering the canyon for photography, which could expose visitors to aerosolized rodent feces.

This case represents the first confirmed instance of SNV infection exported from the United States. Although no clear source of the patient’s exposure was found, it likely occurred during the course of his recreational outdoor activities. Clinicians and public health practitioners should be aware of hantavirus pulmonary syndrome as a potential illness among travelers returning from the southwestern United States. Travelers to this region of the United States should also be informed of the risk factors for hantavirus exposure and methods for risk reduction through public health education materials at popular tourist sites, and tour guide operators should be encouraged to leave the canyon floor undisturbed during their programs.

## Footnotes

* https://www.cdc.gov/hantavirus/surveillance/annual-cases.html.
