# *Erratum:* Vol. 67, No. 40

**DOI:** 10.15585/mmwr.mm6742a7

**Published:** 2018-10-26

In the report “Notes from the Field: Exported Case of Sin Nombre Hantavirus Pulmonary Syndrome – Israel, 2017,” on page 1129, **Maria Morales-Betoulle, PhD; Sara E. Zufan, MPH; and Shannon L.M. Whitmer, PhD,** all affiliated with the Division of High-Consequence Pathogens and Pathology, National Center for Emerging and Zoonotic Infectious Diseases, CDC, should have been included in the author list, and the affiliation for Heather Venkat, DVM, MPH should have been the **Arizona Department of Health Services**. In addition, in the first paragraph, the seventh sentence should have read “**A blood specimen collected on November 9 tested positive for SNV, with an immunoglobulin M titer of ≥1:6400 and an immunoglobulin G titer of ≥1:6400; nested reverse transcription–polymerase chain reaction (RT-PCR) followed by nucleic acid sequencing confirmed the SNV diagnosis**.”

